# Evolution of antibiotic cross‐resistance and collateral sensitivity in *Staphylococcus epidermidis* using the mutant prevention concentration and the mutant selection window

**DOI:** 10.1111/eva.12903

**Published:** 2020-02-25

**Authors:** Natalie Ann Lozano‐Huntelman, Nina Singh, Alondra Valencia, Portia Mira, Maral Sakayan, Ian Boucher, Sharon Tang, Kelley Brennan, Crystal Gianvecchio, Sorel Fitz‐Gibbon, Pamela Yeh

**Affiliations:** ^1^ Department of Ecology and Evolutionary Biology University of California Los Angeles CA USA; ^2^ Department of Molecular, Cell, Developmental Biology University of California Los Angeles CA USA; ^3^ Santa Fe Institute Santa Fe NM USA

**Keywords:** antibiotic resistance, antimicrobial, bacterial evolution, correlated traits, susceptible

## Abstract

In bacteria, evolution of resistance to one antibiotic is frequently associated with increased resistance (cross‐resistance) or increased susceptibility (collateral sensitivity) to other antibiotics. Cross‐resistance and collateral sensitivity are typically evaluated at the minimum inhibitory concentration (MIC). However, these susceptibility changes are not well characterized with respect to the mutant prevention concentration (MPC), the antibiotic concentration that prevents a single‐step mutation from occurring. We measured the MIC and the MPC for *Staphylococcus epidermidis* and 14 single‐drug resistant strains against seven antibiotics. We found that the MIC and the MPC were positively correlated but that this correlation weakened if cross‐resistance did not evolve. If any type of resistance did evolve, the range of concentrations between the MIC and the MPC tended to shift right and widen. Similar patterns of cross‐resistance and collateral sensitivity were observed at the MIC and MPC levels, though more symmetry was observed at the MIC level. Whole‐genome sequencing revealed mutations in both known‐target and nontarget genes. Moving forward, examining both the MIC and the MPC may lead to better predictions of evolutionary trajectories in antibiotic‐resistant bacteria.

## 1 INTRODUCTION

In recent decades, there has been a rapid increase in the prevalence of multi‐antibioticresistant pathogens (Dijkshoorn, Nemec, Nemec, & Seifert, 2007; Leski et al., 2016; Nordmann, Naas, Naas, Fortineau, & Poirel, 2007; Tandogdu et al., 2016; Zalacain et al., 2016). This growing public health threat (Bush et al., 2011; Davies & Davies, 2010; Sanders, 2001; Woolhouse, Waugh, Waugh, Perry, & Nair, 2016) has made it necessary to better understand how evolution of resistance to one antibiotic affects bacterial susceptibility to other antibiotics (Pál, Papp, Papp, & Lázár, 2015). Increased resistance to one antibiotic frequently results in increased resistance to another antibiotic (Haight & Finland, 1952; Obolski, Stein, Stein, & Hadany, 2015; Sanders, 2001), termed cross‐resistance. Conversely, increased resistance to one antibiotic can also often result in decreased resistance to another antibiotic (Obolski et al., 2015; Pál et al., 2015), a phenomenon referred to as collateral sensitivity. By understanding the factors that influence both types of collateral responses, we can better predict evolutionary trajectories of resistant mutants based on the antibiotics they have been exposed to.

There have been hundreds of previous studies on collateral responses, but the vast majority of them have examined these responses only in the context of minimum inhibitory concentration (MIC), which is the antibiotic concentration required to inhibit growth by a set amount (typically 99% inhibition; Barbosa, Beardmore, Beardmore, Schulenburg, & Jansen, 2018; Haight & Finland, 1952; Imamovic & Sommer, 2013; Obolski et al., 2015; Sanders, 2001; Sanders, Sanders, Sanders, Goering, & Werner, 1984; Thomson & Sanders, 1994). A small number of recent studies have started to also examine collateral effects at the mutant prevention concentration (MPC; Imamovic & Sommer, 2013; Podnecky et al., 2018), which is the concentration at which no single‐step resistant mutant can occur (Baquero & Negri, 1997; Bush et al., 2011; Dong, Zhao, Zhao, Domagala, & Drlica, 1999; Drlica, 2003; Drlica & Zhao, 2007). This is often thought of as the concentration needed to prevent the evolution of antibiotic resistance in a typical population size infection of approximately 10^10^ cells (Dong, Zhao, Zhao, Kreiswirth, & Drlica, 2000).

**Figure 1:**
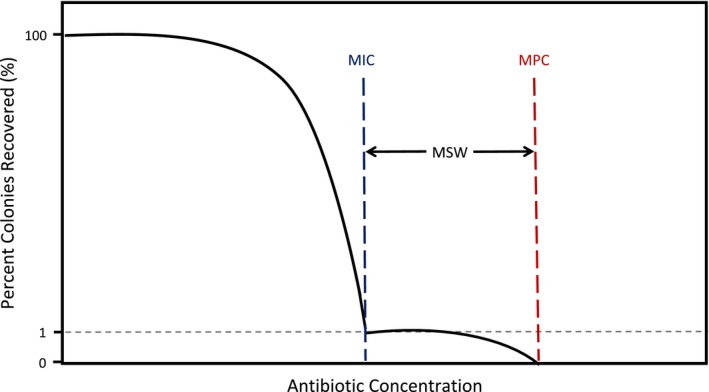
The mutant selection window (MSW) ranges from the MIC to the MPC. The MSW ranges from the MIC (blue line), inhibiting wild‐type growth, to the MPC (red line), at which two simultaneous mutations are needed to survive. The MIC results in a 99% decrease in the numbers of recovered colonies, while MPC results in no recovered colonies. Selection for resistance mutations typically occurs within the MSW. Schematic adapted from Drlica and Zhao (2007)

For example, Imamovic and Sommer (2013) used gentamicin and cefuroxime to show that changes in MPC correlated with collateral responses in resistant mutants in *Escherichia coli*. A few years later, Podnecky et al. (2018) compared the MPC for 17 *E. coli* drug‐strain combinations that showed conserved collateral responses. They found that in 12 of these cases, the change in MPC was consistent with the sign of the collateral responses. Moreover, the mutant selection window (MSW), which is the range of antibiotic concentrations that selects for single‐step resistant mutants (Drlica, 2003; Drlica & Zhao, 2007) and that is bounded by the MIC at the lower end and the MPC at the upper end (Figure 1), was shown to shift up or down depending on the collateral response (Podnecky et al., 2018). Here, we examine networks of collateral responses at both the MIC level and the MPC level, focusing on whether collateral responses are symmetric or asymmetric and how these responses shift the MSW. To investigate these questions, we use 49 drug‐strain combinations of *Staphylococcus epidermidis* (Winslow & Winslow, 1908).

Due to a scarcity of previous work examining the MPC as opposed to the MIC, there is a knowledge gap not only in our understanding of how collateral responses at the MIC and MPC levels differ but also in our understanding of correlated evolution between the MIC and MPC. A review of studies examining the correlation between the MIC and the MPC shows that there tends to be a low positive correlation between these traits (Drlica, Zhao, Zhao, Blondeau, & Hesje, 2006). However, the results have been shown to be species‐dependent based on differing correlations in *E. coli*, *Klebsiella pneumoniae*, *Pseudomonas aeruginosa*, *Staphylococcus aureus*, and *Streptococcus pneumoniae* (Drlica et al., 2006). If the MIC and the MPC are correlated in the data collected here, then selection pressure affecting the MIC could have indirect effects on the MPC for *S. epidermidis* (Brokordt, González, González, Farías, Winkler, & Lohrmann, 2017; Krebs, Feder, Feder, & Lee, 1998; Price & Langen, 1992). The correlations between the MIC and the MPC vary not only with the type of bacteria but also with the type of antibiotics used (Imamovic & Sommer, 2013; Podnecky et al., 2018).

Antibiotics can be categorized into classes based on their mechanisms of action (Chopra & Roberts, 2001; Davis, 1987; Gaynor & Mankin, 2003; Waxman & Strominger, 1983). Cross‐resistance occurs within and across antibiotic classes (Haight & Finland, 1952; Obolski et al., 2015; Sanders, 2001; Thomson & Sanders, 1994). For example, cross‐resistance within the quinolones occurs when the same cellular target has been altered (Martínez & Baquero, 2002; Ruiz, 2003; Sanders, 2001; Sanders et al., 1984). In the case of nalidixic acid‐resistant bacteria, enhanced resistance to ciprofloxacin and norfloxacin is also displayed (Sanders et al., 1984). The resistant mutations to nalidixic acid are described as target modifiers and change the cellular target of the antibiotic to limit its effectiveness (Hemaiswarya, Kruthiventi, Kruthiventi, & Doble, 2008; Martínez & Baquero, 2002). Because of this, these types of mutations are considered effective against antibiotics with similar mechanisms of action (Martínez & Baquero, 2002; Ruiz, 2003; Sanders, 2001; Sanders et al., 1984).

When antibiotics have different mechanisms of action, resistance to one antibiotic does not necessarily cause resistance to another antibiotic. In quinolones, there are cases where resistance to one quinolone does not cause resistance to other quinolones. For example, ciprofloxacin's primary target in *S. pneumoniae* is topoisomerase IV and sparfloxacin's primary target is DNA gyrase. Single‐step mutants selected by one of these antibiotics are less susceptible to the selecting antibiotic but not the other because of different mechanisms of resistance in response to different drug targets (Sanders, 2001).

While different mechanisms of action can sometimes reduce the likelihood of cross‐resistance, this is not always the case. Cross‐resistance across antibiotic classes can occur from mutations in genes that regulate efflux pumps, genes that change outer membrane proteins, or nontargeted mutations in a stress response pathway (Lázár et al., 2014, 2013; Obolski et al., 2015; Sanders et al., 1984). In one case, with quinolone‐resistant *K. pneumoniae*, changes in the outer membrane proteins caused cross‐resistance to beta‐lactams (Sanders et al., 1984). Another study showed that fluoroquinolone‐resistant *E. coli* containing mutations in a topoisomerase gene (*gyr*A) have changed susceptibility of the bacteria to other antibiotics. These changes include increases in resistance to ampicillin, cefoxitin, ciprofloxacin, nalidixic acid, kanamycin, and tobramycin and increases in sensitivity to nitrofurantoin and doxycycline (Lázár et al., 2014).

In addition to cross‐resistance, bacteria can also exhibit collateral sensitivity to antibiotics (Lázár et al., 2013). Since collateral sensitivity occurs when resistance to one drug causes increased susceptibility to other drugs, it is considered an evolutionary trade‐off (Bollenbach, 2015; Pál et al., 2015). For example, cellular uptake of aminoglycosides relies on the proton motive force (PMF). As a result, a reduction in the PMF is frequently the mechanism underlying resistance to aminoglycosides. However, efflux pumps responsible for removing other antibiotics also rely on the PMF. Therefore, resistance to aminoglycosides (due to a reduction in the PMF) can increase susceptibility to other antibiotics, typically expelled through efflux pumps (Pál et al., 2015). In recent years, new resistome studies have demonstrated that the pool of resistance genes is extraordinarily large (Dantas & Sommer, 2014). Characterizing the genomes of the antibiotic‐resistant bacteria examined here is thus important to uncovering new mechanisms of cross‐resistance and collateral sensitivity.

In this study, we ask four main questions. First, is there a correlation between the MICs and MPCs? Second, when resistance to a single antibiotic evolves, how does the MSW change? Third, how do cross‐resistance and collateral sensitivity networks at the MIC level compare to these networks at the MPC level? Is symmetry (i.e., when a strain is resistant to drug A and cross‐resistant to drug B, a strain that is resistant to drug B is also cross‐resistant to drug A) more prevalent at one level than the other? Finally, what are the mutations that are associated with cross‐resistance and collateral sensitivity? To answer these questions for *S. epidermidis*, we used seven antibiotics that covered five different mechanisms of action (Table 1). We spontaneously evolved two resistant mutants per antibiotic, resulting in 14 spontaneous mutant‐resistant strains of *S. epidermidis*. For each of the 14 strains, we determined the MIC, MPC, and thus, the MSW for all seven antibiotics. We then sequenced their genomes and identified mutations affecting resistance.

**Table 1:** A list of antibiotics used and the median and range of the MIC (μg/ml) and MPC (μg/ml) values of the parental strain (*S. epidermidis* ATCC 14990)

Parental strain							
Antibiotics	Abbreviation	MIC			MPC		
		Median	Min	Max	Median	Min	Max
Ciprofloxacin	CPR	0.125	0.125	0.3	1	1	3.75
Doxycycline	DOX	2.6	2	3	16	12	16
Erythromycin	ERY	0.45	0.4	0.5	13	10	16
Gentamicin	GEN	0.293	0.234	0.351	9.36	9.36	9.36
Neomycin	NEO	1	1	1	17.5	15	20
Oxacillin	OX	0.12	0.105	0.12	0.6	0.6	0.6
Tetracycline	TET	8.75	6.25	15	125	125	125

## 2 MATERIALS AND METHODS

### 2.1 Bacteria and antibiotics

We collected spontaneous mutants by evolving *S. epidermidis* (ATCC 14990) to each of the seven antibiotics listed in Table 1 separately. *S. epidermidis* was plated on 150‐mm agar plates with antibiotic concentration ranging from 2 × liquid MIC and ending at 20 × liquid MIC in increments of 2 × liquid MIC estimate. Colonies were selected off the highest concentration where colonies were recovered, in experiments where there was a clear and definable MPC with no colonies recovered after an achieved concentration. We then streak‐purified the colonies from the spontaneous mutant experiments onto separate plates containing antibiotic concentrations above the known MIC to confirm resistance. We inoculated a single colony into LB media (10 g tryptone, 5 g yeast extract, and 10 g NaCl). We then stored this culture in 25% glycerol at −80°C (Mayfield et al., 2013; Taylor & Webster, 2009). We initiated all experiments from a freshly thawed aliquot of this single batch.

We obtained and purified two independent spontaneously resistant mutants for each antibiotic, resulting in 14 resistant strains. The resistant strains were named based off of the antibiotic used to select for them. For example, the two strains resistant to ciprofloxacin were labeled as CPR R1 and CPR R2. We termed these “spontaneous mutant‐resistant strains.”

We further independently evolved *S. epidermidis* (ATCC 14990) to each of the seven antibiotics in Table 1. We evolved eight strains to each antibiotic for about 100 generations, resulting in 56 adapted resistant strains. We evolved the strains in a step‐wise manner where the antibiotic concentration was continually doubled from ½ × MIC to 8 × MIC every 48 hr over the course of 10 days. We termed these “adapted resistant strains.” This was done to capture the possibility of mutation acquisition being dependent on the dose of antibiotic the bacteria were exposed to (Jahn, Munck, Munck, Ellabaan, & Sommer, 2017; Lindsey, Gallie, Gallie, Taylor, & Kerr, 2013; Oz et al., 2014).

### 2.2 Liquid MIC

We obtained MICs for the parental *S. epidermidis* ATCC 14990 strain and all 70 resistant strains (spontaneous and adapted) for every antibiotic assessed in this study. We created a liquid culture using 2 ml of LB in a culture tube and adding 150 µl of the thawed cell culture aliquot. We then placed this tube in a shaker set at 220 revolutions per minute (RPM) and 37°C to incubate until the OD_600_ reached 0.3 (Tecan Infinite M200 PRO Multimode Microplate Reader). We loaded fresh LB media and the selected antibiotic at varying concentrations into a 96‐well plate to have a volume of 100 µl per well. We diluted bacterial cultures by a factor of 1:500 to create the inoculum. We added 100 µl of the inoculum to each well resulting in a final volume of 200 µl per well. We measured bacterial growth by reading the OD after 18 hr and defined the MIC as the minimum antibiotic concentration observed to inhibit growth by at least 95% among all replicate wells. We included both positive (LB + bacteria) and negative (LB only) controls on each plate to ensure bacterial growth of the particular strain and no contamination of media. We used these measurements to obtain a rough estimate of the MIC to determine MIC in agar, as described below.

### 2.3 Agar MIC and MPC assays

#### 2.3.1 Bacterial preparation

We prepared the cultures from a single freezer aliquot (250 µl) by inoculating into 10 ml of LB. We grew the cultures overnight for 18 hr at 37°C and 160 RPMs. Afterward, we inoculated the entire bacterial culture into 450 ml of fresh LB until an OD600 between 0.45 and 0.70 was reached. Then at 4°C, we centrifuged the cultures at about 3,000 *g* for 10 min to obtain a high concentration of cells when plating and set aside the supernatant. We re‐suspended the pellet in 7.5 ml of the original supernatant (Figure S1A).

#### 2.3.2 Determining agar MIC

Because there may be discrepancies between the liquid MIC estimate and agar MIC, we measured MIC in agar simultaneously with MPC experiments. Since identical increments were taken in each biological replicate, little variation would arise due to the liquid MIC estimate. The liquid MIC and agar MIC only differed slightly (±0.5 μg/ml) when increments of at least twofold were used. We prepared agar plates using 1,000 ml of autoclaved Milli‐Q water with 15 g agar powder and one 25 g LB tablet (10 g tryptone, 5 g yeast extract, 10 g NaCl, and 1.5 g/L Tris/Tris‐HCl).

To determine MIC, we plated 100‐mm petri plates with 20 ml of LB agar with antibiotics ranging from 0.2 × liquid MIC estimate to 1.7 × liquid MIC estimate in increments of 0.1 × liquid MIC estimate (Figure S1B). We inoculated each of these plates with 10^5^ CFU via sterile glass beads following the Copacabana method (Mills, Gareau, & Garcia, 2005; Worthington, Luo, & Pelo, 2001) and included a positive control containing no antibiotic. We incubated the plates at 37°C for 72 hr, and colonies were counted. We used two replicates, and following another study (Tan et al., 2009), we defined the MIC in agar as the first antibiotic concentration where the number of colonies was reduced by 95% or greater from the control in both of the two plates. While many studies use the 99% cutoff (Haight & Finland, 1952; Obolski et al., 2015; Sanders, 2001; Thomson & Sanders, 1994), we used a slightly lower cutoff to account for random noise in the data. For each drug‐strain combination, we determined the MIC in three separate instances resulting in six plates. We recorded the median and range for each MIC.

#### 2.3.3 MPC determination and analysis

To determine the MPC, we used three 150‐mm plates with 60 ml of LB agar for each antibiotic concentration ranging from 2 × liquid MIC estimate and ending at 20 × liquid MIC estimate in increments of 2 × liquid MIC estimate (Figure S1C). We then inoculated the plates with 10^10^ CFUs via sterile glass beads following the Copacabana method (Worthington et al., 2001). We defined the MPC as the lowest antibiotic concentration where there was no growth across all three replicates (Allen, Kaatz, Kaatz, & Rybak, 2004; Dong et al., 1999; Drlica, 2003; Drlica & Zhao, 2007; Firsov, Lubenko, Lubenko, Smirnova, Strukova, & Zinner, 2008; Firsov et al., 2003; Hansen, Zhao, Zhao, Drlica, & Blondeau, 2006; Metzler et al., 2004). We conducted the MPC assays in triplicates resulting in a total of nine agar plates per drug‐strain combination. We calculated both the median and the range.

### 2.4 Mutant selection window

With the MIC and MPC values determined, we measured the MSW in terms of the parental MIC value. This allowed us to directly compare how the MSW changes across multiple strains.

### 2.5 Whole‐genome sequencing

We performed whole‐genome sequencing on the parental strain of *S. epidermidis* ATCC 14990 and on all spontaneous mutant‐resistant strains. The sequences were paired‐end with a length of 150 bp. We aligned the sequences to the *S. epidermidis* ATCC 12228‐reference genome to elucidate the genetic changes underlying their antibiotic‐susceptibility phenotypes. We used *S. epidermidis* ATCC 12228 as the reference genome due to its more complete gene annotation. We streak‐purified all strains on LB agar plates prior to DNA library preparation and *HiSeq* sequencing at the Genewiz Next Generation Sequencing facility in South Plainfield, New Jersey. We note that most of the plasmids in the reference genome, *S. epidermidis* ATCC 12228, are not represented in the *S. epidermidis* ATCC 14990 strains. However, the smallest plasmid, NC_005008 (4,439 bp), is fully represented as a circular element in all strains and carries a tetracycline resistance gene and two replication protein genes (Putonti et al., 2017). Sequencing coverage shows most strains have five copies of this plasmid. However, DOX R1, DOX R2, and TET R2 appear to have 12–16 copies (Tables S2 and S3). One of the parental strains (parental strain 2) appears to have lost the plasmid and has one tenth of the main chromosome coverage. We suspect this may be due to the plasmid being lost during cultivation for sequencing for parental strain 2.

### 2.6 Bioinformatics analysis

We removed the adapter sequences from sequence reads, and the quality was checked using Trim Galore! (http://www.bioinformatics.babraham.ac.uk) with quality trimming turned off. Trim Galore! is a wrapper for cutAdapt (Martin, 2011) and FastQC (https://www.bioinformatics.babraham.ac.uk). We mapped trimmed reads using BWA‐MEM v.0.7.12‐r1039 (Li & Durbin, 2010) to the *S. epidermidis* ATCC 12228 genome (2,499,279 bp chromosome & 6 plasmids, 4,439–24,365 bp, NCBI Accessions NC_004461.1 and NC_005003‐8). All samples had at least 97% of the adapter trimmed reads mapped to the ATCC 12,228 genome. We performed variant discovery and filtering with GATK v 3.7‐0‐gcfedb67 (McKenna et al., 2010), including MarkDuplicates, HaplotypeCaller in GVCF mode with ploidy 1, GenotypeGVCFs, and finally VariantFiltration with the following hard filters applied: QD < 20.0, MQ < 40.0, FS > 60.0, SOR > 3.0, MQRankSum < −12.0, ReadPosRankSum < −8.0. SnpEff (Cingolani et al., 2012) was used to determine the context of the variants and predict the functional impact. We removed variants with an allele frequency of 1 across all of the strains including the two parent strains with GATK's SelectVariants. We used the VCFtools package (Danecek et al., 2011) to inspect summaries of the filter's effects and the transition transversion ratios for each.

After manual inspection of alignments, we excluded additional variants from regions highly divergent from the reference genome, as the alignments in these regions are unreliable mainly due to structural rearrangements. These excluded regions are main chromosome positions 37885‐38551, 57541‐57702, 91802‐93606, 200225, 666092, 1519681‐1519683, 2311095‐2312854, and 2471276‐2471507. We used GATK's DepthOfCoverage to determine mean depth of coverage across each sample and across each genomic element (Tables S2 and S3).

## 3 RESULTS

### 3.1 Correlated evolution of the MIC and MPC

**Figure 2:**
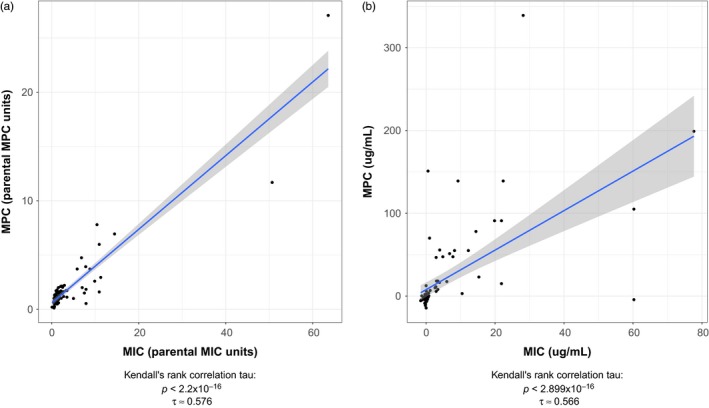
A positive correlation is found between MIC and MPC in *Staphylococcus epidermidis*. The MIC is plotted against the MPC in (a) parental MIC and parental MPC units (e.g., MIC_strain_/MIC_parent_ and MPC_strain_/MPC_parent_) and (b) μg/ml. A positive correlation was found (Kendall rank correlation test, [a] *τ* = .576, *p* < .001, [b] *τ* = .566, *p* < .001)

We found an increase in the median MIC and the median MPC (compared with the parental strain) for all 14 spontaneous mutant‐resistant strains of *S. epidermidis* (Table 1) except TET R2. For both the MIC and the MPC for this strain, we were unable to determine values due to an extremely high level of resistance. Kendall's rank correlations of the MIC and the MPC data were used to evaluate any possible relationship between the MIC and MPC due to the data heteroscedasticity. The overall correlation of the MICs and MPCs showed that as the MIC increased, the MPC increased (*τ* = .5510332, *p* < 2.2 × 10^–16^; Figure 2). This trend holds true when examining each individual spontaneous mutant‐resistant strain across all antibiotics using Kendall's rank correlation (*p* < .05 for each strain), with the exception of doxycycline and tetracycline (Figure S2).

**Figure 3:**
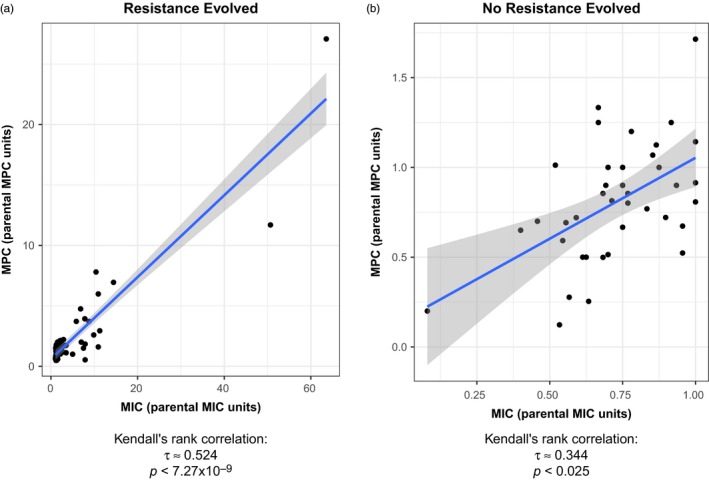
The positive correlation found between MIC and MPC weakens if cross‐resistance has not evolved. The fold change after evolution of the MIC medians (MIC_strain_/MIC_parent_) is plotted against the fold change after evolution of the MPC medians (MPC_strain_/MPC_parent_) for (a) spontaneous mutant‐resistant strains that showed evolved resistance at the MIC (MIC fold change >1; Kendall rank correlation test, *τ* = .524, *p* < .001) and (b) spontaneous mutant‐resistant strains that did not show evolved cross‐resistance at the MIC (MIC fold change ≤1). A positive correlation between the change in the MIC and the change in the MPC was found in both instances, but the correlation was weaker when cross‐resistance did not evolve (Kendall rank correlation test, *τ* = .344, *p* < .025)

We observed that the outcomes of evolution affected this correlation. If resistance evolved, through direct selection or through cross‐resistance, the correlation remained roughly the same as the overall correlation between all MICs and MPCs (*τ* = .5238549, *p* < 7.3 × 10^–9^; Figure 3a). However, if no cross‐resistance evolved, observed through no change in the MIC or through instances of collateral sensitivity, the correlation between MIC and MPC became weaker (*τ* = .3438369, *p* < .025; Figure 3b).

The mixture of bactericidal and bacteriostatic antibiotics used could have confounded the relationship between MIC and MPC. Bactericidal drugs are ciprofloxacin, oxacillin, and gentamicin, and bacteriostatic drugs are doxycycline, erythromycin, and tetracycline. We found no difference in the size of the MSW and no difference in the fold change in MIC or MPC between bactericidal and bacteriostatic drugs. Neomycin has both bactericidal and bacteriostatic activities so we left it out of our analysis.

### 3.2 Changes in the mutant selection window

**Figure 4:**
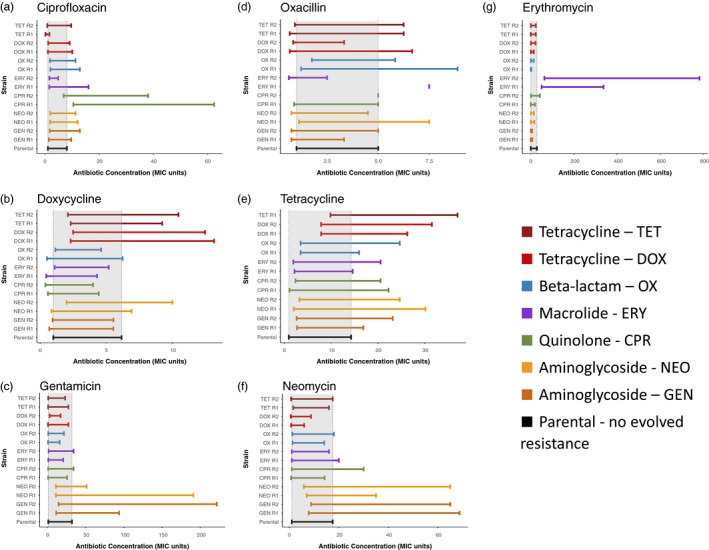
The MSW tends to shift to the right and widen as resistance evolves. The gray regions indicate the mutant selection windows of the parental strain. The MSW for each spontaneous mutant‐resistant strain is shown in panels (a–g), which are divided by the antibiotic used to determine the MSW. As resistance evolves, the MSW tends to shift to the right and widen as compared to the parental strain (gray‐shaded region). When cross‐resistance does not evolve, the MSW is highly variable. In Panel (d), ERY R1 and CPR R2 have MSWs that appear as single points because the median MIC and median MPC for these strains are the same, so the MSW has a size of zero. Given the large antibiotic concentration increments used in this study, it is very likely that the true values lie in between the increments. In Panel (e), the TET R2 MSW is missing because the MIC and MPC for tetracycline of the TET R2 were undetermined due to high levels of resistance

We compared the MSWs using the median MIC and the median MPC for the parental and spontaneous mutant‐resistant strains across all antibiotics (Figure 4). When resistance evolved, regardless of whether it was through direct resistance to drug X or through cross‐resistance, the MSW shifted right and widened. Paired *t* tests were used to evaluate both the increase in the MIC (*p* < .0005) indicating the right shift and the increase in range of the MSW (*p* < .05) indicating the widening of the MSW. This was a general trend of the MSW and is seen when resistance is selected for or when cross‐resistance evolves either within an antibiotic class (i.e., gentamicin/neomycin and tetracycline/doxycycline) or across classes (i.e., DOX R1 and TET R1 exposed to oxacillin). However, when there is no evolved cross‐resistance or when there are cases of collateral sensitivity at the MIC, the MSW does not follow the trend of shifting right and widening. In these cases, the MSW either narrows or behaves in a highly variable way.

For example, when treated with erythromycin, only the ERY R1 and ERY R2 strains had a larger MIC and wider MSW. All other spontaneous mutant‐resistant strains treated with erythromycin appeared to have MSWs that narrowed or were unchanged when compared to the parental strain (Figure 4g). We showed that collateral sensitivity to erythromycin at the MPC level frequently occurred, while the MIC was essentially not affected (Figure 4g). This means that the MSW for erythromycin narrowed for most of the spontaneous mutant‐resistant strains other than erythromycin‐resistant ones.

Another exception to the pattern of the MSW widening and shifting right appeared for strains treated with oxacillin (Figure 4d). Of these strains, only OX R1 and OX R2 consistently showed resistance and a widening of the MSW. The MSW of all other spontaneous mutant‐resistant strains treated with oxacillin seems to vary in size dramatically and has no consistent trend.

### 3.3 MPC cross‐resistance and collateral sensitivity

**Figure 5:**
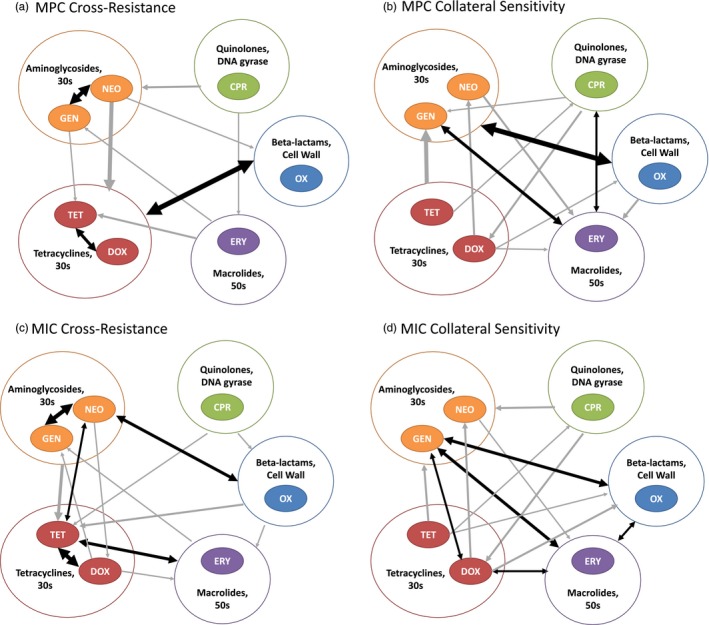
Symmetrical relationships are more prevalent at the MIC level than at the MPC level. This figure design is based on Pál et al. (2015) showing the network maps of the types of MPC and MIC cross‐resistance and collateral sensitivity. Arrows represent the presence, amount, and direction of the outcomes. Arrows originate at the selective antibiotic of a resistant strain and end at the antibiotic susceptibility being tested. Black double arrows highlight symmetrical relationships. Arrows may originate and end at the larger circles encompassing one to two antibiotics; this indicates all respective strains or antibiotics exhibit the same relationships. The weight of each arrow indicates the number of outcomes exhibiting the same relationship. (a) MPC cross‐resistance patterns. (b) MPC collateral sensitivity patterns. (c) MIC cross‐resistance patterns. (d) MIC collateral sensitivity patterns. Both cross‐resistance and collateral sensitivity were identified both within and across antibiotic classes. The collateral response networks show similar patterns at the MIC and MPC levels, but the MIC level has notably more symmetry (five symmetrical cross‐resistances and five symmetrical collateral sensitivities) than the MPC level (three symmetrical cross‐resistances and three symmetrical collateral sensitivities)

To investigate instances of cross‐resistance and collateral sensitivity, we used the MPC ranges to create a network map of the types of cross‐resistance (Figure 5, Table 1 and Table S1). We define cross‐resistance as a rightward shift in the range of the spontaneous mutant‐resistant strains, where these strains and the parental strain ranges do not overlap (max_parent_ < min_resistant strain_). Collateral sensitivity is a downward shift in the range of the spontaneous mutant‐resistant strains, where these strains and the parental strain ranges do not overlap (max_resistant strain_ < min_parent_).

Cross‐resistance was observed a total of 25 times and at least once in each spontaneous mutant‐resistant strain (Figure 5a). Cross‐resistance was found in both of the spontaneous mutant‐resistant strains (R1 and R2) 64% of the time for the same antibiotic. We found cross‐resistance to antibiotics within and across different classes (Figure 5a). Patterns of cross‐resistance among antibiotics of the same class have already been observed at the MIC level (Sanders et al., 1984), and most of these patterns are preserved when considering the MPC values (Figure 5).

Collateral sensitivity was found in both spontaneous mutant‐resistant strains (R1 and R2) 62% of the time. Regarding collateral sensitivity, our main findings were as follows: (a) collateral sensitivity to erythromycin and gentamicin was common (Figure 5b), and (b) resistance to doxycycline was generally associated with collateral sensitivity to nontetracycline antibiotics (neomycin, gentamicin, oxacillin, and erythromycin; Figure 5b).

The adapted resistant strains showed extremely high cross‐resistance to all antibiotics, and the MICs for these adapted resistant strains were so high that they exceeded the maximum solubility for some of the antibiotics used (Please see the supplemental information for more detailed methods and results; Appendix S1 and Figure S1).

Next, we asked whether there were any cases of symmetrical cross‐resistance and/or symmetrical collateral sensitivity and if the resulting networks were similar at the MIC and MPC levels. A symmetrical relationship is defined as having the same type of cross‐resistance for each set of resistant strains to their complimentary antibiotic. For example, a symmetrical relationship would occur if a strain is resistant to antibiotic A and cross‐resistant to antibiotic B, and a different strain is resistant to antibiotic B and cross‐resistant to antibiotic A. Cases of symmetrical cross‐resistance and collateral sensitivity can be viewed as a positive feedback loop. Symmetrical cross‐resistance positively reinforces resistance to either antibiotic, whereas symmetrical collateral sensitivity positively reinforces susceptibility to either antibiotic.

We found that symmetrical relationships were more prevalent at the MIC level (five cross‐resistant and five collaterally sensitive symmetries) than at the MPC level (three of each symmetry type; Figure 5). We identified two possible symmetrical relationships of cross‐resistance within the same antibiotic classes of tetracyclines (tetracycline and doxycycline) and aminoglycosides (neomycin and gentamicin), both of which were observed at the MIC and MPC level (Figure 5a,c). We also identified two possible symmetrical relationships between classes: an MPC cross‐resistance symmetry between the tetracyclines (tetracycline and doxycycline) and the beta‐lactam (oxacillin) and an MPC collateral sensitivity symmetry between the aminoglycosides (neomycin and gentamicin) and the beta‐lactam (oxacillin). Both of these symmetrical relationships between classes were only constantly observed at the MPC level (Figure 5a,b).

### 3.4 Mutations in the genome

We found thirteen unique antibiotic resistance mutations. Nine were missense mutations, and the remaining four consisted of disruptive in‐frame insertions, mutations in the upstream region, changes in plasmid copy number, or stop codons (Table 2). Resistance typically occurs through mutations within a target gene. The spontaneous mutant‐resistant strains CPR R1, CPR R2, ERY R1, ERY R2, GEN R1, GEN R2, NEO R1, and NEO R2 all gained mutations in genes that are associated with resistance to their respective antibiotic (Besier, Ludwig, Ludwig, Brade, & Wichelhaus, 2003; Bodley, Zieve, Zieve, Lin, & Zieve, 1969; Chittum & Champney, 1995; Davydova, Streltsov, Streltsov, Wilce, Liljas, & Garber, 2002; Sreedharan, Peterson, Peterson, & Fisher, 1991). We found instances of resistance that may be due to novel or nontarget mutations (SE_p103, SE0706, SE608, SE1860, SE2021) and are shared between both strains (Table 2).

**Table 2:** Resistance conferring mutations

Resistance	Associated gene or plasmid	Strains	Type of mutation	References	Chromosomal position	Nucleotide substitution	Amino acid substitution
Ciprofloxacin	*SE1037*	CPR R1	Missense	Sreedharan et al. (1991)	1045114	C → A	p.Ser80Tyr
		CPR R2		Ferrero, Cameron, and Crouzet (1995)		C → T	p.Ser80Tyr
Erythromycin	*rpIV*	ERY R1 ERY R2	Disruptive in‐frame insertion	Chittum and Champney (1995) Davydova et al. (2002)	1863928	G → GCTTGTACGTTTATTAATTGCA	p.Ser101_His102insAlaIleAsnLysArgThrSer
Aminoglycoside	*fusA*	GEN R1	Missense	Bodley et al. (1969)	310912	G → T	p.Arg59Leu
		GEN R2	Missense		312466	C → A	p.Ser577Tyr
		NEO R1 NEO R2	Missense	Besier et al. (2003)	312253	A → C	p.Tyr506Ser
Tetracycline	*NC_005008*	TET R2	Change in plasmid copy number	Putonti et al. (2017)	n/a	n/a	n/a
	*SE0075*	TET R1 TET R2	Stop gained	*	74230	C → T	p.Gln281*
	*SE2021*	TET R1 TET R2	Missense	*	2059908	G → C	p.Met201Ile
Doxycycline	*SE_p103*	DOX R1 DOX R2	Upstream gene variant	*	3147	T → C	n/a
	*SE0706*	DOX R1 DOX R2	Missense	*	696607	G → A	p.Arg70Lys
	*SE608*	DOX R1 DOX R2	Missense	*	595362	G → T	p.Ala85Ser
	*SE1860*	DOX R1 DOX R2	Missense	*	1898875	G → A	p.Ala238Val
Oxacillin	*SE2021*	OX R1 OX R2	Missense	*	2059908	G → C	p.Met201Ile

* Mutations that have not yet been identified and/or confirmed in the literature.

## 4 DISCUSSION

### 4.1 Effects of resistance on MIC and MPC

Previous research has yielded much information about collateral responses measured using MICs (Haight & Finland, 1952; Imamovic & Sommer, 2013; Obolski et al., 2015; Sanders, 2001; Sanders et al., 1984; Thomson & Sanders, 1994). Here, we examined whether and how the MIC and the MPC are related, how the MSW changes as resistance evolves, and what the patterns of collateral responses at the MPC level are.

The widespread correlation between the MIC and the MPC (Figure 2) in the spontaneous resistant strains suggested that as selection acts on the MIC, indirect selection occurs at the MPC level in *S. epidermidis*. This is consistent with previous work correlating these concentrations in other bacterial species (Drlica et al., 2006). Intriguingly, our results suggest that evolution of resistance affects that correlation. We find that the overall positive correlation of the MIC and MPC is strongly held when resistance is evolved (*τ* = .5238549, *p* < 7.3 × 10^–9^) but becomes substantially weaker when cross‐resistance has not evolved (*τ* = .3438369, *p* < .025; Figure 3).

That is, if the collateral result of resistance evolution does not increase the MIC, the correlation weakens. Since the overall correlation is relatively strong as MIC increases, we expect and observe that the MPC increases as well. But if the MIC decreases, there is a much lower likelihood that the MPC will decrease as well. Although there is still a significant correlation in the cases where the collateral result of resistance evolution does not increase the MIC, this positive correlation is seen only about 30% of the time (Figure 3b). It is important to note that the correlation between MIC and MPC (using all cases where resistance evolved and where it did not evolve) is not significant for tetracycline and doxycycline (Figure S3), underscoring the importance of testing this correlation between each antibiotic–bacteria combination.

Our observed pattern of the MSW generally shifting has also been observed in *E. coli* (Podnecky et al., 2018). However, it has not previously been reported in the context of collateral responses that the MSW shifts and widens. This pattern may be important for understanding the effects of aggressive treatment strategies like using high drug dosages (Read, Day, Day, & Huijben, 2011). Reducing bacterial load through these strategies can make it easier for a patient's immune system to defeat an infection and decrease the probability of de novo mutations that confer resistance from arising (Drlica, 2003; Read et al., 2011). However, if highly resistant mutants already exist within the original infection or if de novo mutants arise that are highly resistant, aggressive antibiotic treatment applies the strongest possible selection for these mutants. This gives highly resistant mutants the best possible chance of repopulating the infection and spreading to other people (Drlica, 2003; Read et al., 2011). Our finding that the MSW shifts right and widens as resistance evolves provides important context for this work. It suggests that when high concentrations of an antibiotic are used, the range of concentrations that selects for resistant mutants generally increases and makes the resulting mutants even more resistant (Drlica, 2003).

Oz and colleagues further demonstrated the implications of high antibiotic concentrations on resistance using isogenic *E. coli* populations. In their study, they evolved two populations under strong selection and two populations under mild selection for each of 22 antibiotics over 3 weeks. Upon constructing cross‐resistance networks, they found that bacterial populations that had evolved antibiotic resistance under strong selection demonstrated higher levels of cross‐resistance than those that had evolved antibiotic resistance under milder selection (Oz et al., 2014). Our result is consistent with their finding: Mutants selected at the MPC level generally displayed MSWs that widened and shifted to the right when exposed to other antibiotics. Taken together, these findings suggest that combination drugs are likely to be more effective than ever‐increasing dosages of a single drug when considering the role that selective pressure can have on collateral effects (Oz et al., 2014) and the size of the resulting MSWs (Michel, Yeh, Chait, Moellering, & Kishony, 2008).

### 4.2 Cross‐resistance and collateral sensitivity at the network level

We found that there are more symmetrical relationships at the MIC level than at the MPC level. The MPC symmetries tended to be a subset of the MIC symmetries. This may be because spontaneous mutant‐resistant strains were originally selected at the MIC level, and although MIC and MPC are positively correlated, the MPC did not always increase with the MIC. In cases where cross‐resistance did not evolve, or where there was collateral sensitivity, the MPC did not increase along with the MIC and the symmetrical relationships were not preserved at the MPC level. Additionally, the correlation between MIC and MPC was not perfect and varied depending on the antibiotic (Figure S2), so this also contributed to MIC symmetrical relationships not always carrying over to the MPC level.

Our finding of symmetrical MPC cross‐resistance within tetracyclines and the aminoglycosides (Figure 5a) and MPC cross‐resistance between different antibiotic classes is congruent with previous work conducted using MICs (Pál et al., 2015). For example, it has been shown that *E. coli* K12 strains resistant to tetracycline or chloramphenicol exhibited a decreased sensitivity to fluoroquinolones (Cohen, McMurry, McMurry, Hooper, Wolfson, & Levy, 1989), and our findings at the MPC level support this.

Our results at the MPC level for collateral sensitivity (Figure 5b) also support results from a previous study that used the MIC values to find cases of collateral sensitivity across antibiotics with various mechanisms of action in *E. coli* (Lázár et al., 2014). Our findings make sense when viewed in light of studies showing that collateral responses are relatively stable as resistance develops (Munck, Gumpert, Gumpert, Wallin, Wang, & Sommer, 2014). Recent work suggests that collateral sensitivity and cross‐resistance may be even more important than drug interactions when it comes to using drug combinations to combat resistance (Munck et al., 2014; Rodriguez de Evgrafov, Gumpert, Gumpert, Munck, Thomsen, & Sommer, 2015). This is because drug interaction types change as resistance develops but the mechanisms behind collateral responses are more stable (Munck et al., 2014; Rodriguez de Evgrafov et al., 2015).

For example, a study examining six antibiotics and five antibiotic pair combinations found no relationship between drug interaction type and resistance evolution beyond wild‐type levels of resistance, but found that cross‐resistance and collateral sensitivity were important in predicting resistance evolution (Rodriguez de Evgrafov et al., 2015). Upon examining the genomes of *E. coli* that were evolved in the presence of five different antibiotics and the resulting 10 antibiotic pairs, it was found that collaterally sensitive drug combinations consistently created environments in which mutants resistant to either antibiotic were counterselected, and thus, there was decreased evolution of resistance overall (Munck et al., 2014).

### 4.3 Genes involved in resistance

We found that some spontaneous mutant‐resistant strains had mutations within the same genes, yet show distinct phenotypic variation. For example, TET and OX spontaneous mutant‐resistant strains conferred an identical mutation on *SE2021*, an amino acid transporter gene (Zhang et al., 2003), yet have phenotypic differences in the MSW in the presence of doxycycline (Table 2 and Figure 4). The MSW of TET shifts to the right and widens compared with the MSW of OX, which remains the same as the wild‐type MSW (Figure 4b).

Additionally, DOX R1 and DOX R2 were genetically identical, but we observed a case where DOX R1 was exposed to oxacillin and the strain showed MPC collateral sensitivity against one drug (oxacillin), while DOX R2 showed MPC collateral sensitivity against a different drug (erythromycin). Differing responses of cross‐resistance and collateral sensitivity among replicates have been observed in other experiments (Barbosa et al., 2017), and whole‐genome sequencing revealed distinct evolutionary paths of resistance in these cases (Barbosa et al., 2017). Since the liquid MIC for oxacillin was determined to be 0.08 µg/ml, the low MIC value may have affected the accuracy of measuring the MPC in this case. The MPC, unlike the MIC, is not a single value but could vary significantly due to Luria‐Delbruck fluctuations (Gianvecchio et al., 2019; Jones, Thomas, Thomas, & Rogers, 1994; Luria & Delbrück, 1943).

Despite this variation in MPC values, we generally found that patterns of the types of cross‐resistance are common within antibiotic classes at both the MIC and MPC levels, which may be attributed to the types of mutations they share. For example, both aminoglycoside resistance strains, GEN and NEO (R1 and R2), had different mutations on the same gene *fusA*, a ribosomal gene originally identified as conferring resistance to fusidic acid (Table 2; O'Neill, Larsen, Larsen, Henriksen, & Chopra, 2004). GEN and NEO spontaneous mutant‐resistant strains showed similar phenotypic responses across the seven drugs even though the individual mutations resulted in an amino acid change in different locations within *fusA*. Studies have shown that there are many different mutations within *fusA* that result in resistance to fusidic acid and have similar MICs (Laurberg et al., 2000), yet the specific amino acid substitutions that we have identified here have not previously been reported. However, *fusA* has also been reported as encoding for an elongation factor that is responsible for increased resistance in both *E. coli* (Zengel, Archer, Archer, & Lindahl, 1984) and *T. thermophilus* (Laurberg et al., 2000). We suspect that this characteristic could also play a role in the resistance phenotypes of the GEN and NEO spontaneous mutant‐resistant strains of *S. epidermidis*. Although we did not look into other traits, such as fitness costs, associated with these genomic changes, we believe that future work can help explain the observed phenotypic variation. Further genomic characterizations can help identify more genetic mechanisms underlying cross‐resistance and collateral sensitivity (Hickman, Munck, Munck, & Sommer, 2017).

### 4.4 Potential clinical applications

Since the MSW typically broadens under antibiotic treatment, this suggests that typical treatment strategies, which use antibiotic concentrations well above the MIC based on the antibiotic's pharmacokinetic and pharmacodynamics values (Bonhoeffer, Lipsitch, Lipsitch, & Levin, 1997; Levison & Levison, 2009), can potentially select for mutations that confer greater resistance. This indicates the limited utility of using ever‐increasing dosages of a single drug to narrow the MSW.

Notably, we found that when there was no evolved cross‐resistance or when there were cases of collateral sensitivity at the MIC, the MSW did not follow the trend of shifting right and widening. In the case of oxacillin‐treated strains, only OX R1 and OX R2 consistently showed resistance and a widening of the MSW. All other strains had MSWs that did not follow a consistent trend (Figure 4d). When examining this figure, it is important to note that ERY R1 and CPR R2 have MSWs that appear as single points because their median MIC and median MPC were the same, resulting in MSWs of size zero. Variation within Figure 4d highlights the importance of testing each antibiotic to understand its effect on resistant strains rather than assuming that all antibiotics will cause the MSW to shift and widen.

Another interesting case of the MSW not following this general trend occurred with the erythromycin‐treated strains. Here, the MSW narrowed or stayed nearly the same for all spontaneous mutant‐resistant strains except erythromycin‐resistant ones (Figure 4g). Even though resistance to erythromycin can become extremely strong, it may be a good option for the treatment of infections that are already resistant to another antibiotic. For these infections, there would be a reduced chance of subsequently evolving cross‐resistance to erythromycin, as evidenced by the narrowed range of concentrations in which further single‐step resistant mutants could evolve in our experiments. It is interesting to note that the MSW of CPR R2 widened slightly in response to erythromycin rather than narrowing like the MSW of other strains (Figure 4g). The deviation from this general trend may be due to the difference in the point mutation in SE1037 within the CPR spontaneous mutant‐resistant strains (Table 2). Antibiotics that tend to gain collateral sensitivity in the MPC and to shrink the MSW, such as erythromycin, may be a good component for an antibiotic cycling therapy or combinational therapy.

Collateral sensitivity and cross‐resistance are frequently observed not only in the laboratory but also in clinical settings. For example, a study examining resistance in 2,478 *E. coli* isolates from urinary tract infections found high levels of cross‐resistance between many pairs of drugs, including gentamicin and ampicillin, ciprofloxacin and sulfamethoxazole, and trimethoprim and sulfamethoxazole (Kahlmeter & Menday, 2003). Separate work that also examined resistance in *E. coli* isolates from urinary tract infections used 16 antibiotics and observed 141 instances of cross‐resistance (e.g., between ciprofloxacin and chloramphenicol and between nitrofurantoin and amoxicillin) and 92 instances of collateral sensitivity (e.g., between ciprofloxacin and gentamicin and between ciprofloxacin and colistin; Podnecky et al., 2018).

Clinicians can potentially take advantage of collateral sensitivity through antibiotic cycling or combination therapy. Cycling between antibiotics that demonstrate collateral sensitivity may prevent the fixation of mutations that result in stronger resistance to one antibiotic, and may also result in hypersensitivity to other antibiotics (Imamovic & Sommer, 2013). Our findings of potential symmetrical collaterally sensitive relationships suggest two‐drug sets of antibiotics to use in further investigations of the antibiotic cycling strategy, including oxacillin and gentamicin. For example, oxacillin may initially be effective at killing a bacterial population, but with repeated exposure, resistance to this drug will likely evolve. If the bacterial population is then treated with gentamicin and evolves resistance to this new drug, it may become susceptible to oxacillin again. This type of antibiotic cycling strategy, that is, taking advantage of collateral sensitivity, may help extend the usefulness of currently available antibiotics (Bush et al., 2011; Davies & Davies, 2010; Gonzales et al., 2015; Imamovic & Sommer, 2013; Sanders, 2001).

However, when considering a cyclic approach to treating bacterial infections, it is also important to take into consideration our finding that the MPC does not correlate as strongly to the MIC, and thus, the MSW does not behave in a predictable way when cross‐resistance does not evolve for spontaneous mutant‐resistant strains. Since cyclic treatment strategies depend on resistance to new drugs not evolving due to collateral sensitivity (Imamovic & Sommer, 2013), the MPC should be evaluated for each step of the cycle. This could help ensure that dosage concentrations are not within the new MSW to account for cases in which the MSW widens even if the MIC decreases.

Our results can expand on the cycling strategy by identifying potential cases of symmetrical collateral sensitivity using the MSW across seven antibiotics that span five classes. Symmetrical cases of collateral sensitivity can be much more useful than asymmetrical ones, because the order in which a population of bacteria evolves resistance matters less, since there is collateral sensitivity in both directions (Imamovic & Sommer, 2013). Due to the small number of replicates we use here and evidence that collateral sensitivity patterns in laboratory strains do not always apply to clinical isolates (Imamovic & Sommer, 2013), it is important to conduct further studies using clinical isolates. Furthermore, bacteria are not typically selected at MPC concentrations in clinical settings because the toxicity resulting from such high concentrations is too much for the human body to handle (Blondeau, Zhao, Zhao, Hansen, & Drlica, 2001; Gianvecchio et al., 2019; Metzler et al., 2004).

In conclusion, we have shown how the mutant prevention concentration (MPC) and the mutant selection window (MSW) change for a range of drugs after the evolution of resistance to one antibiotic in *S. epidermidis*. When examining our data for each spontaneous mutant‐resistant strain, we found that the MSWs tend to shift right and widen as antibiotic resistance evolves, showing a strong correlation between the MIC and MPC. However, the MSW varies dramatically and the correlation between the MIC and MPC weakens when cross‐resistance has not evolved at the MIC. When examining our data at the network level, we found that cross‐resistance and collateral sensitivity patterns within MIC and MPC networks are similar, and there are more cases of symmetrical relationships at the MIC level than at the MPC level. Our genetic analysis of the strains used here further supports the importance of traditional target‐gene mutations and reveals possible novel or nontarget mutations in antibiotic resistance evolution. Overall, using both the MIC and the MPC to evaluate antibiotic resistance may lead to better predictions of the evolutionary outcomes of resistant mutants when exposed to different antibiotics.

## CONFLICT OF INTEREST

None declared.

## Data Availability

The phenotypic assay data for this study are available in the supporting information, and the whole‐genome sequencing data are available on NCBI's SRA (Accession Number: PRJNA593298).
