# Supplementary figures and images for: Evolution of antibiotic cross‐resistance and collateral sensitivity in Staphylococcus epidermidis using the mutant prevention concentration and the mutant selection window
Source: Evol Appl. 2020 Feb 25;13(4):808–23. doi: 10.1111/eva.12903 (PMC7086048; doi:10.1111/eva.12903)

A

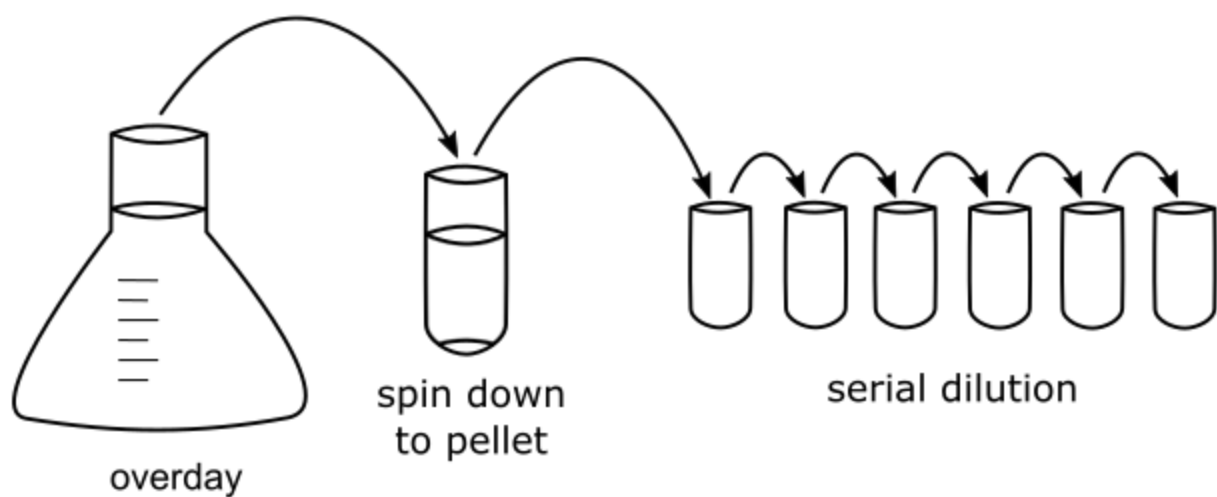

B

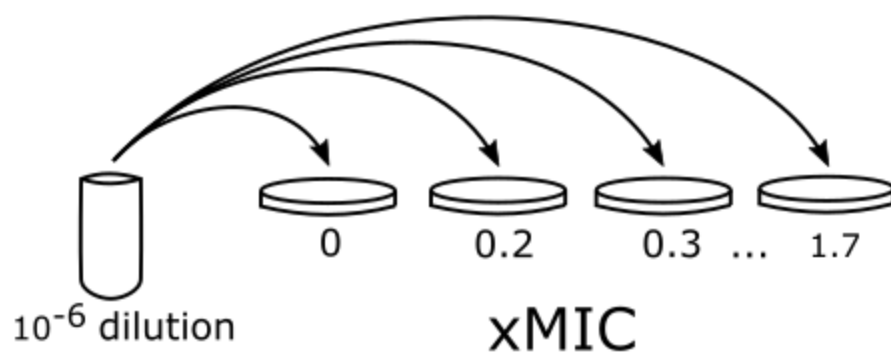

C

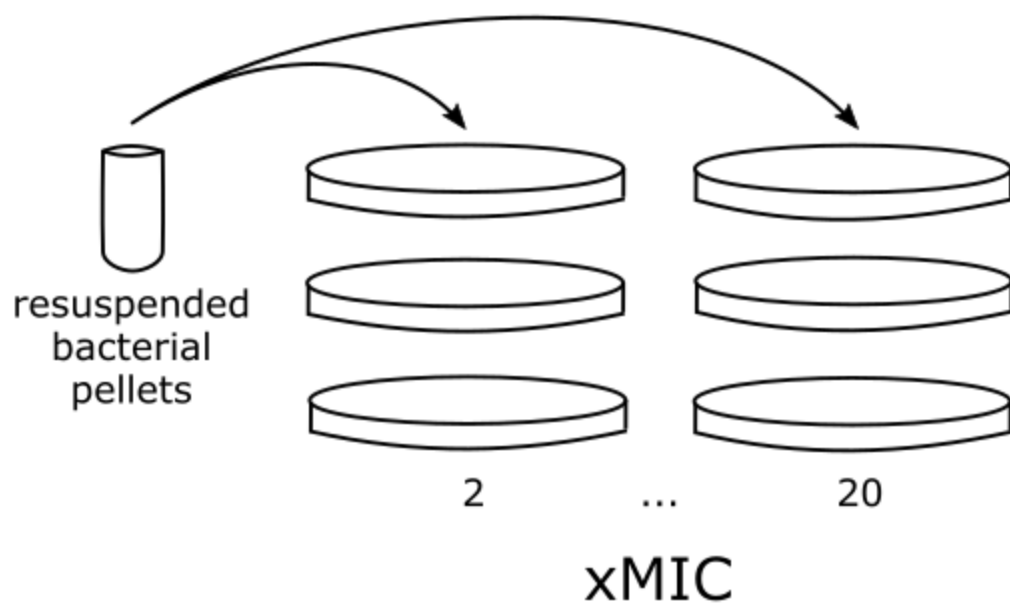

Supplement: Supplementary file 1 [file EVA-13-808-s001.pdf]

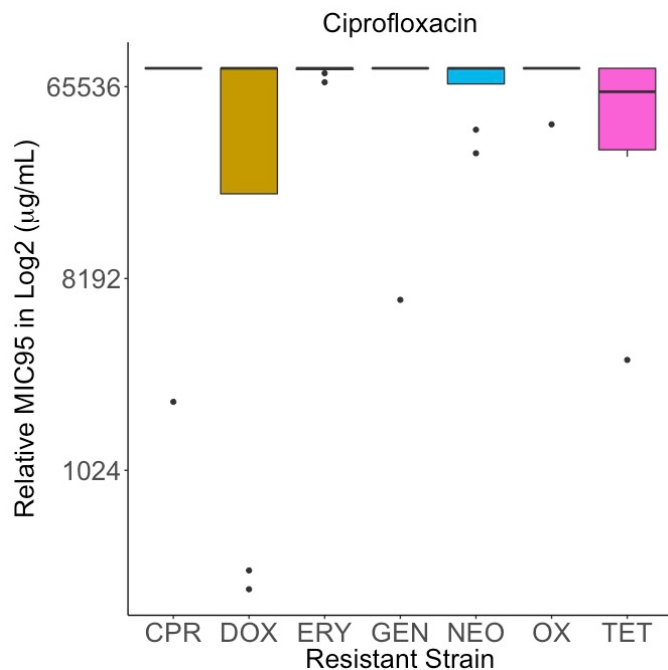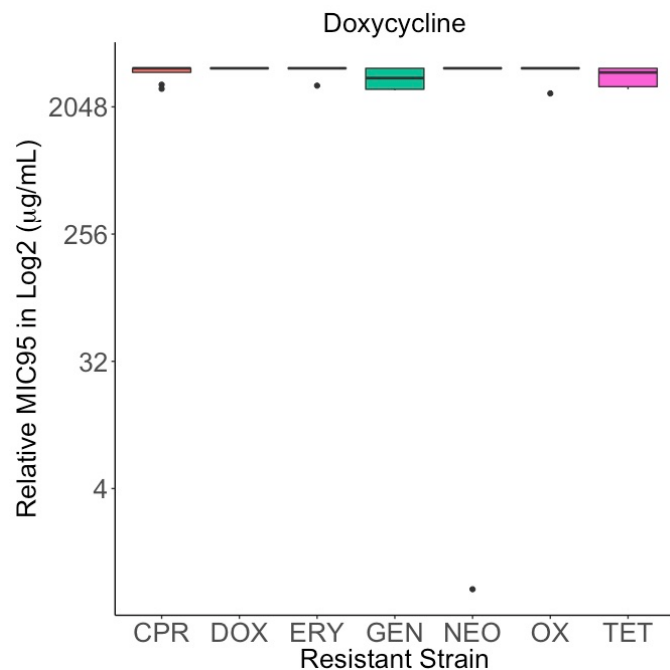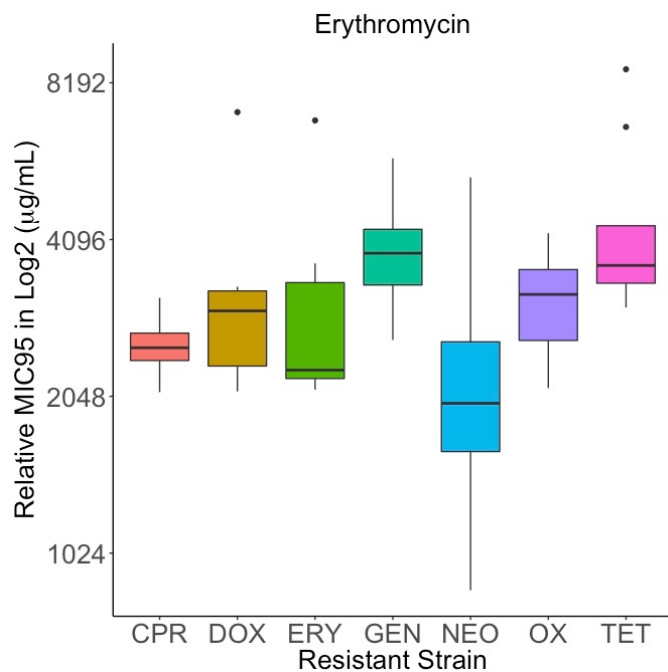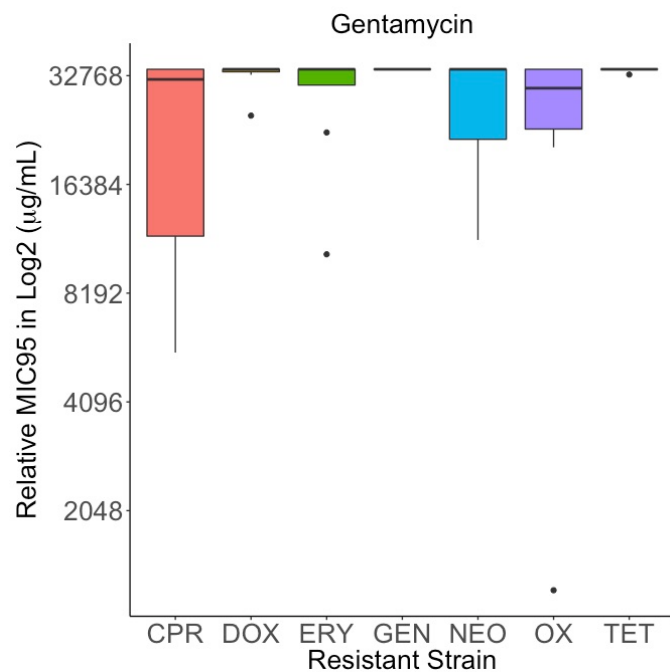

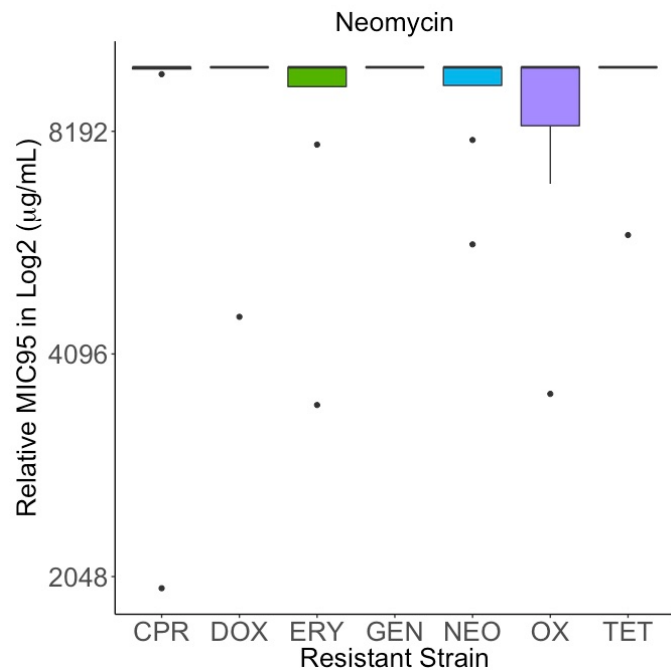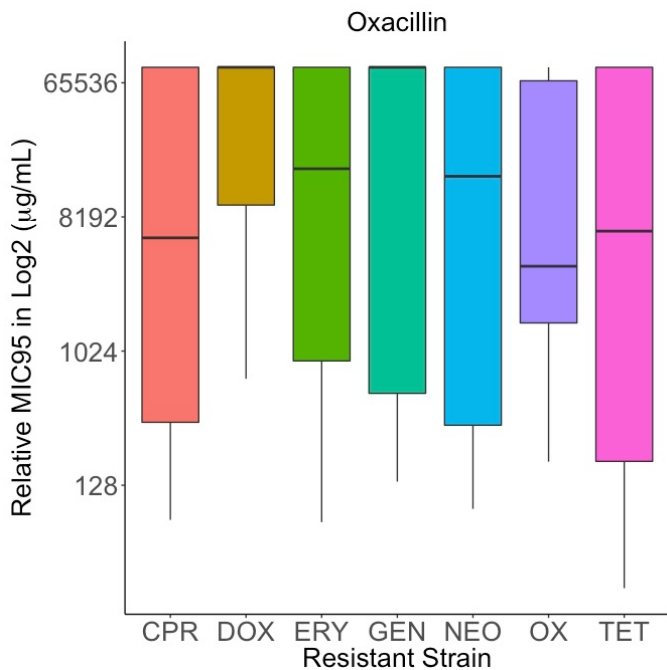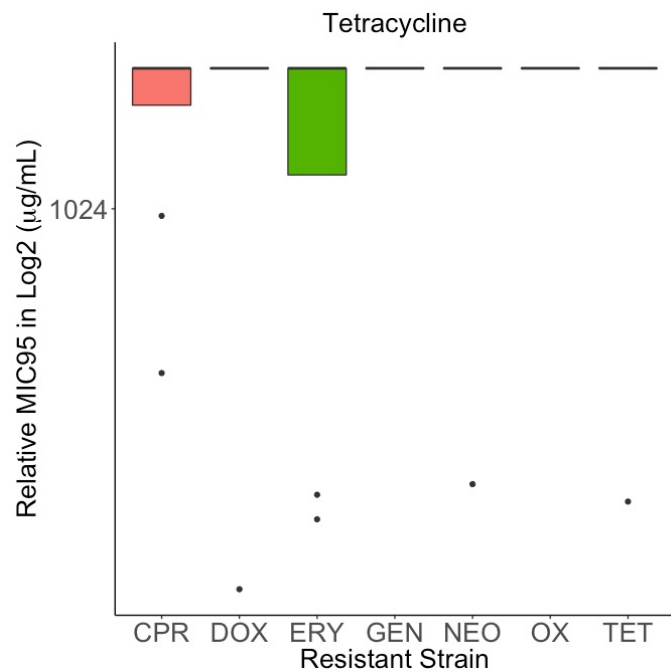

Supplement: Supplementary file 3 [file EVA-13-808-s003.pdf]
