# Supplementary material for: Evolution of antibiotic cross‐resistance and collateral sensitivity in Staphylococcus epidermidis using the mutant prevention concentration and the mutant selection window
Source: Evol Appl. 2020 Feb 25;13(4):808–23. doi: 10.1111/eva.12903 (PMC7086048; doi:10.1111/eva.12903)
Supplement: Supplementary file 2 [file EVA-13-808-s002.pdf]

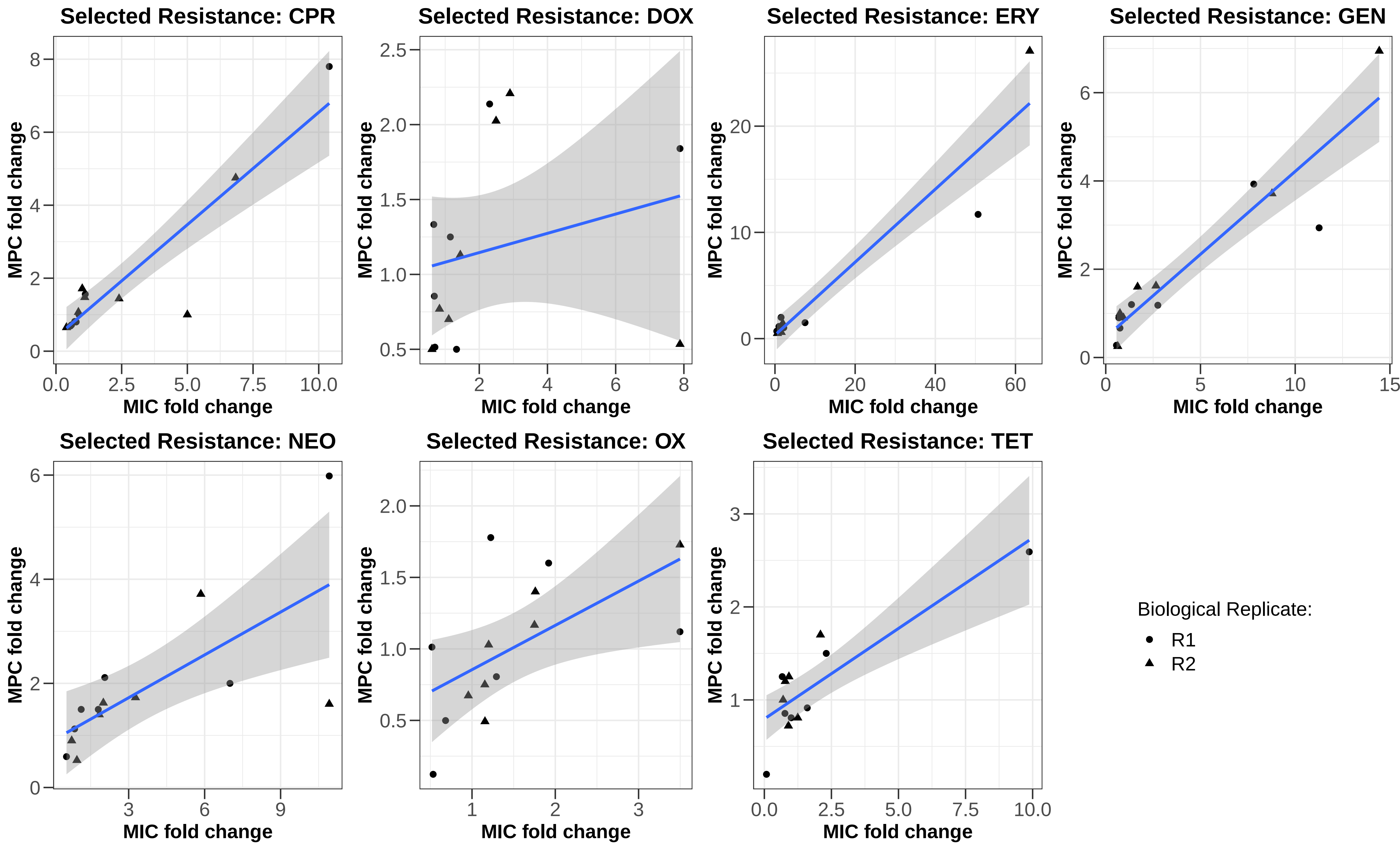
Selected Resistance: CPR
Selected Resistance: DOX
Selected Resistance: ERY
Selected Resistance: GEN
Selected Resistance: NEO
Selected Resistance: OX
Selected Resistance: TET
MPC fold change
MIC fold change
Biological Replicate:
R1
R2
