# Supplementary material for: Evolution of antibiotic cross‐resistance and collateral sensitivity in Staphylococcus epidermidis using the mutant prevention concentration and the mutant selection window
Source: Evol Appl. 2020 Feb 25;13(4):808–23. doi: 10.1111/eva.12903 (PMC7086048; doi:10.1111/eva.12903)
Supplement: Supplementary file 4 [file EVA-13-808-s004.pdf]

Spontaneous Mutant Resistant Strains

strain	Antibiotic	MIC			MPC			strain	Antibiotic	MIC			MPC		
		median	min	max	median	min	max			median	min	max	median	min	max
	CRP	1.3	1.04	1.3	7.8	7.28	7.8		CRP	0.855	0.76	1.045	4.75	3.42	4.75
	DOX	1.536	1.152	1.92	11.52	7.68	11.52		DOX	1.04	0.78	1.04	10.4	10.4	10.4
	ERY	0.25	0.15	0.4	9	9	9		ERY	0.493	0.384	0.548	19.18	19.18	21.92
CPR R1	GEN	0.225	0.225	0.225	7.5	7.5	7.5	CPR R2	GEN	0.25	0.2	0.3	10	10	10
	NEO	0.713	0.713	0.95	14.25	9.5	23.75		NEO	1	1	1	30	25	30
	OX	0.105	0.105	0.12	0.6	0.6	0.6		OX	0.6	0.6	0.9	0.6	0.6	0.9
	TET	9.75	7.8	11.7	195	117	195		TET	21	18	27	180	120	240
	CRP	0.19	0.175	0.225	2	2	2.5		CRP	0.195	0.195	0.195	0.6	0.6	0.6
	DOX	1.19	0.98	1.4	11.2	11.2	14		DOX	2.85	2.85	2.85	13.5	12	15
	ERY	22.8	18.24	25.84	152	121.6	152		ERY	28.6	28.6	28.6	352	172	352
ERY R1	GEN	0.3	0.3	0.3	6	6	7.5	ERY R2	GEN	0.4	0.4	0.4	10	10	10
	NEO	1	0.75	1	20	20	20		NEO	1	1	1	16	16	16
	OX	0.9	0.075	0.9	0.9	0.6	1.5		OX	0.075	0.075	0.075	0.3	0.3	0.6
	TET	19.2	16	22.4	128	128	192		TET	17	17	17	180	180	180
	CRP	0.225	0.225	0.225	1.5	1.5	1.5		CRP	0.23	0.21	0.23	1.4	1.4	1.75
	DOX	2.25	2.25	2.25	18	18	18		DOX	5.2	5.2	5.2	26	26	26
	ERY	0.245	0.245	0.245	7.7	7.7	7.7		ERY	0.43	0.34	0.51	6.8	6.8	8.5
NEO R1	GEN	3.2	3.2	3.2	56	56	56	NEO R2	GEN	3.2	3.2	3.2	15	15	15
	NEO	7	7	7	35	35	35		NEO	5.85	3.25	5.85	65	52	91
	OX	0.135	0.135	0.135	0.9	0.9	0.9		OX	0.09	0.07	1	0.54	0.54	0.54
	TET	18	18	18	264	264	264		TET	28.6	23.4	28.6	216	216	216
	CRP	0.01	0.01	0.01	0.2	0.15	0.25		CRP	0.0975	0.075	0.12	1.2	1.2	1.2
	DOX	6	4.8	7.2	24	34	36		DOX	5.44	5.44	7.48	27.2	27.2	27.2
	ERY	0.45	0.45	0.6	10.5	10.5	13.5		ERY	0.56	0.525	0.6	10.5	9	12
TET R1	GEN	0.225	0.2	0.225	8	7	8	TET R2	GEN	0.263	0.225	0.3	6.75	4.5	9
	NEO	1.6	1.6	1.8	16	12	24		NEO	0.7	0.525	0.875	17.5	10.5	17.5
	OX	0.08	0.08	0.08	0.75	0.75	1		OX	0.11	0.075	0.1375	0.75	0.5	1.25
	TET	86.4	86.4	86.4	324	324	324		TET	liq	liq	liq	>200	200	200
	CRP	0.125	0.125	0.163	1.25	1.25	1.25		CRP	0.138	0.125	0.15	1.13	1	1.25
	DOX	5.99	5.13	6.84	34.2	34.2	34.2		DOX	6.48	5.94	7.02	32.4	32.4	32.4
	ERY	0.6	0.6	0.6	6.5	6	7		ERY	0.65	0.50	0.8	10	10.00	14
DOX R1	GEN	0.2	0.2	0.2	8	8	8	DOX R2	GEN	0.85	0.70	1	5	4.00	6
	NEO	0.7	0.6	0.9	6	6	12		NEO	0.613	0.525	0.7	8.75	7	10.5
	OX	0.08	0.07	0.09	0.8	0.8	0.8		OX	0.1	0.1	0.1	0.4	0.4	0.4
	TET	69	69	69	230	230	230		TET	69	69	69	276	276	276
	CRP	0.24	0.24	0.24	1.6	1.2	2		CRP	0.22	0.22	0.22	1.4	1.2	1.6
	DOX	1.35	1.35	1.35	16.2	16.2	18.9		DOX	3	3	3	12	12	12
	ERY	0.24	0.24	0.24	1.6	1.6	1.6		ERY	0.52	0.52	0.52	6.4	4.8	6.4
OX R1	GEN	0.2	1-0	0.2	4.67	4-5	5	OX R2	GEN	0.28	0.28	0.28	6.3	6.3	6.3
	NEO	1.293	1.175	1.41	14.1	9.4	14.1		NEO	1.2	1.2	1.2	18	18	24
	OX	0.147	0.12	0.16	1.067	0.8	1.6		OX	0.21	0.21	0.21	0.7	0.7	0.7
	TET	30.6	30.6	30.6	140	140	180		TET	30.6	30.6	30.6	216	216	252
	CRP	0.17	0.16	0.18	1.2	1.2	1.2		CRP	0.21	0.2	0.22	1.6	1.6	1.6
	DOX	1.8	1.8	1.8	14.4	14.4	14.4		DOX	2.43	2.16	2.7	14.4	14.4	14.4
	ERY	0.255	0.24	0.27	3.6	3.6	3.6		ERY	0.285	0.27	0.3	3.3	2.4	4.2
GEN R1	GEN	3.3	3.3	3.3	27.5	22	33	GEN R2	GEN	4.23	3.9	4.55	65	65	65
	NEO	7.813	6.25	9.375	68.75	50	87.5		NEO	8.775	7.8	9.75	65	65	78
	OX	0.09	0.08	0.1	0.4	0.4	0.4		OX	0.09	0.08	0.1	0.6	0.6	0.6
	TET	24.05	20.35	27.75	148	111	185		TET	23.2	21.75	24.65	203	203	203
