# Supplementary material for: Evolution of antibiotic cross‐resistance and collateral sensitivity in Staphylococcus epidermidis using the mutant prevention concentration and the mutant selection window
Source: Evol Appl. 2020 Feb 25;13(4):808–23. doi: 10.1111/eva.12903 (PMC7086048; doi:10.1111/eva.12903)
Supplement: Supplementary file 5 [file EVA-13-808-s005.pdf]

Target	Total Coverage	Average Coverage	CPR R1	CPR R2	DOX R1	DOX R2	TET R1	TET R2	NEO R1	NEO R2	GEN R1	GEN R2	ERY R1	ERY R2	OX R1	OX R2	parental 1	parental 2
NC_004461.1:1-2499279	60403517981	24168.38	1427.71	1440.08	1578.9	1467.91	1441.01	1580.67	308.87	1493.61	1723.12	1338.39	1801.43	387.47	1729.66	340.33	1711.43	1536.14
NC_005008.1:1-4439	674233319	151888.56	7511.31	6159.05	20524.63	18334.83	6490.4	25178.53	1251.98	6380.82	8391.02	7027	8893.61	2221.88	9847.82	1811.94	7127.48	77.05
NC_005007.1:1-4679	287200	61.38	3.66	4.71	3.86	3.37	4.37	4.72	0.52	3.89	2.96	3.78	4.24	1.06	4.13	0.75	4.84	3.17
NC_005003.1:1-6585	115736	17.58	1.16	0.97	0.94	0.9	1.03	1.64	0.02	1.75	0.93	1.25	1.13	0	1.23	0.01	1.31	0.92
NC_005006.1:1-8007	116	0.01	0.01	0	0	0	0	0	0	0	0	0	0	0	0	0	0	0
NC_005005.1:1-17261	193088089	11186.38	651.68	901.27	617.12	638.02	856.7	831.13	145.82	553.68	525.59	650.1	722.73	214.9	905.28	163.74	745.01	613.13
NC_005004.1:1-24365	53498254	2195.7	133.92	158.2	122.24	128.23	166.23	147.32	25.04	123.86	146.15	134.11	154.88	33.39	151.82	28.65	144.13	119.19
