# Supplementary material for: Evolution of antibiotic cross‐resistance and collateral sensitivity in Staphylococcus epidermidis using the mutant prevention concentration and the mutant selection window
Source: Evol Appl. 2020 Feb 25;13(4):808–23. doi: 10.1111/eva.12903 (PMC7086048; doi:10.1111/eva.12903)
Supplement: Supplementary file 7 [file EVA-13-808-s007.docx]

**Supporting Information Figure Legends**

**Supplemental Figure 1.** **Overview of the MPC assay procedure. A)** The cell culture is grown, centrifuged down, re-suspended, and diluted to the desired concentration. **B)** MIC is determined through inoculation of a 10^-6^ dilution on 100 mm plates. The lowest concentration inhibiting at least 95% of growth relative to the control (no antibiotic) plate is considered to be the MIC. **C)** MPC is determined through inoculation of approximately 10^10^ CFUs on larger 150mm plates. The MPC value is considered to be the lowest concentration resulting in no growth.

**Supplemental Figure 2.** **Correlation of the MIC and MPC Tends to Be High Across Selected Resistance Groups.** The fold change after evolution of the MIC medians (MIC_strain_/MIC_parent_) is plotted against the fold change after evolution in the MPC medians (MPC_strain_/MPC_parent_). The shapes represent which strain the median was determined for; circles are R1 and triangles are R2. A Kendall’s rank correlation test was performed for each set of spontaneous mutant resistant strains: CPR$(\tau=0.729, p<2 x {10}^{-4})$, DOX $(\tau=0.322, p>0.05)$, ERY $(\tau=0.552, p<0.01)$, GEN $(\tau=0.789, p<9.678 x {10}^{-5})$, NEO $\left( \tau=0.7, p<0.001 \right),$ OX $\left( \tau=0.575, p<0.005 \right),$ TET $(\tau=0.39, p>0.05)$. Even though the correlations between MIC and MPC are significant overall, they vary by antibiotic. For the tetracyclines (DOX and TET), the correlations are not significant.

**Supplemental Figure 3.** **Adapted Resistant Strains Show High Levels of Cross-Resistance to All Antibiotics.** Relative MIC_95_ values of adapted resistant strains are shown to be very high. Resistant strains are presented in the x-axis, grouped according to antibiotic of selected resistance. Data for the eight biological replicates was grouped by the antibiotic of selected resistance. The relative MIC μg/mL (observed MIC/ancestral MIC) is presented on the y-axis in a Log2 scale. Any MIC value that exceeded the threshold of 10,000μg/mL was assigned the value of 10,000μg/mL. MIC values for the adapted resistant strains were close to or above solubility thresholds, making MPC experiments with these strains not feasible.

**Supporting Information Table Titles and Notes**

**Supplemental Table 1.** Median and ranges of MIC (μg/mL) and MPC (μg/mL) of all spontaneous mutant resistant strains. Bolded text indicates instances of resistance. Red bold indicates cross-resistance, determined by non-overlapping ranges. Blue italicized indicates collateral sensitivity, as determined by non-overlapping ranges, when compared to parental strains.

**Supplemental Table 2.** Mean coverage across the main chromosome and plasmids.

**Supplemental Table 3.** Plasmid coverage divided by the main chromosome coverage.

**Supplemental Appendix 1. Liquid MIC Estimates for Independently Evolved Strains.**

We obtained estimates of the MICs for the 56 independently evolved resistant strains for every antibiotic assessed in this study. We created a liquid culture using 2mL of LB in a culture tube and adding 150µL of the thawed cell culture aliquot. We then placed this tube in a shaker set at 220 revolutions per minute (RPM) and 37°C to incubate until the OD_600_ reached 0.3 (Tecan Infinite M200 PRO Multimode Microplate Reader). We loaded fresh LB media and the selected antibiotic at varying concentrations into a deepwell 96-well plate to have a volume of 200µL per well. We diluted bacterial cultures by a factor of 1:500 to create the inoculum. We added 200µL of the inoculum to each well resulting in a final volume of 400µL per well. After 18hrs we aspirated, transferred 200ml of each well into a 96-well plate, and measured bacterial growth by reading the OD. We defined the MIC as the minimum antibiotic concentration observed to inhibit growth by at least 95% amongst all replicate wells. We included both positive (LB + bacteria) and negative (LB only) controls on each plate to ensure bacterial growth of the parental strain and no contamination of media. We used these measurements to obtain a rough estimate of the MIC to determine MIC in agar, as described in the methods.
