# Supplementary material for: Evolution of antibiotic cross‐resistance and collateral sensitivity in Staphylococcus epidermidis using the mutant prevention concentration and the mutant selection window
Source: Evol Appl. 2020 Feb 25;13(4):808–23. doi: 10.1111/eva.12903 (PMC7086048; doi:10.1111/eva.12903)
Supplement: Supplementary file 6 [file EVA-13-808-s006.pdf]

Target	CPR R1	CPR R2	DOX R1	DOX R2	TET R1	TET R2	NEO R1	NEO R2	GEN R1	GEN R2	ERY R1	ERY R2	OX R1	OX R2	parental 1	parental 2
NC_005008.1:1-4439	5.3	4.3	13.0	12.5	4.5	15.9	4.1	4.3	4.9	5.3	4.9	5.7	5.7	5.3	4.2	0.1
NC_005007.1:1-4679	0.0	0.0	0.0	0.0	0.0	0.0	0.0	0.0	0.0	0.0	0.0	0.0	0.0	0.0	0.0	0.0
NC_005003.1:1-6585	0.0	0.0	0.0	0.0	0.0	0.0	0.0	0.0	0.0	0.0	0.0	0.0	0.0	0.0	0.0	0.0
NC_005006.1:1-8007	0.0	0.0	0.0	0.0	0.0	0.0	0.0	0.0	0.0	0.0	0.0	0.0	0.0	0.0	0.0	0.0
NC_005005.1:1-17261	0.5	0.6	0.4	0.4	0.6	0.5	0.5	0.4	0.3	0.5	0.4	0.6	0.5	0.5	0.4	0.4
NC_005004.1:1-24365	0.1	0.1	0.1	0.1	0.1	0.1	0.1	0.1	0.1	0.1	0.1	0.1	0.1	0.1	0.1	0.1
